# A Rare Case of Clavicle Osteomyelitis in a Child and Literature Review

**DOI:** 10.1155/2016/8252318

**Published:** 2016-12-05

**Authors:** Elisavet-Anna Chrysochoou, Charalampos Antachopoulos, Konstantinos Badekas, Emmanuel Roilides

**Affiliations:** ^1^Infectious Diseases Unit, 3rd Department of Pediatrics, Medical Faculty, Aristotle University School of Health Sciences, Hippokration Hospital, Thessaloniki, Greece; ^2^Department of Pediatrics, General Hospital of Serres, Serres, Central Macedonia, Greece

## Abstract

Acute clavicle osteomyelitis in children is rare representing <3% of osteomyelitis cases. We treated a 12-year-old boy who presented with acute pain in the right clavicle and high fever for 4 days. MRI showed abnormal signal in the right clavicle with periosteal reaction.* Staphylococcus aureus* isolated from blood was susceptible to methicillin, clindamycin, and macrolides. Clindamycin was given intravenously for 3 wks and orally for another 3 wks with no recurrence. We reviewed clavicle osteomyelitis cases in children searching PubMed English literature. From a total of 89 studies retrieved, only 6 fulfilled the criteria and were analyzed. Sixteen patients (56% female) were included with a median age of 9 yrs (range 2 wks–16 yrs). Osteomyelitis was hematogenous in most cases, with* S. aureus* being the most frequent cause, isolated from either blood or tissue. Symptoms included fever, swelling, and localized bone tenderness. Antimicrobial therapy lasted for 4–12 weeks (median 7.5). Three patients required drainage or curettage. Recurrence occurred in 1/16 cases (6.2%) and persistence of symptoms occurred to 2/16 cases (12.5%) reported before 90s with unknown antimicrobial susceptibility of the pathogen. Acute clavicle osteomyelitis mainly affects older children and has generally good prognosis.* Staphylococcus aureus* is most commonly implicated and surgery may be needed.

## 1. Introduction

Osteomyelitis is inflammation of bones located in the metaphysis and is more frequent in the lower limbs [1]. The diagnosis of osteomyelitis in childhood is usually straightforward and timely use of appropriate antimicrobial therapy has virtually eliminated mortality and long-term sequelae. However, the incidence of chronicity, deformity, and disability is substantial when diagnosis and management are delayed or incorrect. In 65–75% of cases the femur, tibia, or humerus is involved. Involvement of other long bones is less common and of bones such as clavicles, ribs, spine, and bones of the hands and feet is unusual; thus, at these sites, diagnostic problems may present. Among these bones, the clavicle is involved in 1–3% [2].

The clavicle is a unique bone. It is the first bone to ossify in the human embryo and is the only long bone to ossify intramembranously [3–6]. Most lesions of the clavicle are traumatic and pose few diagnostic difficulties. Nontraumatic clavicular lesions, on the other hand, are rare and frequently present problems in diagnosis [7]. We herein report a case of clavicle osteomyelitis in a child and review published cases of acute clavicle osteomyelitis in pediatric patients.

## 2. Patients and Methods

We describe a case of clavicle osteomyelitis diagnosed and treated in our pediatric departments. In addition, we reviewed the PubMed English literature on clavicle osteomyelitis in children. We used the terms “osteomyelitis”, “clavicle”, and “children” as key words. We included those cases for which the diagnosis of acute osteomyelitis of clavicle, the age (until 16 years old), and the gender of patients were reported. We excluded chronic cases of clavicle osteomyelitis and those cases for which the age and the gender of patients were unknown. From each case of acute osteomyelitis of clavicle analyzed, we recorded the age, the gender, the symptoms and signs, the side of clavicle affected, the microorganism isolated, the treatment (kind of antibiotic, route and duration of administration, surgical drainage, or curettage), and the outcome (cure, recurrence, or persistence).

## 3. Case Presentation

Α 12-year-old boy was admitted in the Department of Pediatrics of Serres General Hospital with acute pain in the right clavicle and high fever (39-40°C) for four days. Two episodes of vomiting and one episode of diarrhea were reported during admission but did not recur. Clinical examination revealed mild swelling, redness, and intense pain upon pressure on the sternal end of the right clavicle, which extended to the supraclavicular fossa and caused torticollis.

**Figure 1:**
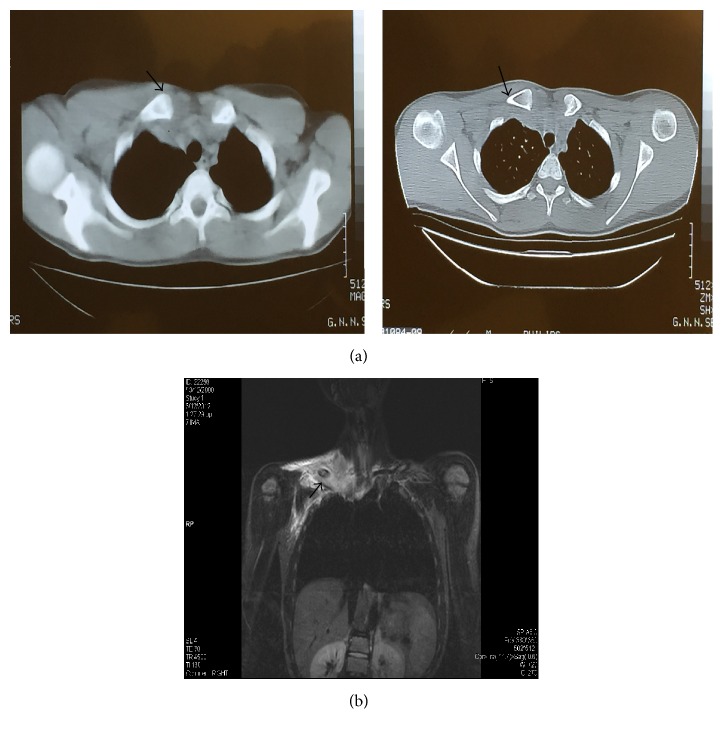
(a) The CT scan showed widening of the soft tissues and the proximal part of the right clavicle without obvious change in the bone marrow and without erosion of the cortex. (b) The MRI showed abnormal signal in the right clavicle with periosteal reaction.

Laboratory tests were WBC 9770/*μ*L (neu 81.6%, lymph 12.1%), PLT 162,000/*μ*L, Hb 14.8 g/dL, Hct 43.1%, and CRP 8.5 mg/dL. An X-ray of the clavicle was unremarkable. A CT scan showed widening of the sternoclavicular joint with swelling of the muscular tissue in the ipsilateral area (Figure 1(a)). After blood cultures were taken and with a possible diagnosis of osteomyelitis, intravenous antimicrobial treatment was empirically initiated with vancomycin and cefotaxime. The child was then transferred to the 3rd Department of Pediatrics, Hippokration Hospital of Thessaloniki, for further evaluation and treatment.

An MRI scan performed at Hippokration Hospital showed abnormal signal in the right clavicle, mainly in its sternal end with periosteal reaction; moreover, the signal was intensely abnormal in the muscles and the soft tissue of the supraclavicular and subclavian fossa with diminished diffusion and intense enrichment of the above areas (Figure 1(b)). These findings suggested osteomyelitis with a presence of a small abscess in the sternal end of the clavicle, with coexistence of extensive pyomyositis (Figure 1(b)).

On the 3rd day,* Staphylococcus aureus* was isolated from blood culture, susceptible to methicillin, macrolides, and clindamycin. Treatment was then changed to clindamycin. The patient defervesced on the 5th day of hospitalization and gradual clinical and laboratory improvement was observed in the subsequent 1-2 weeks. The treatment was continued with intravenous antibiotics for a total of 3 weeks and oral clindamycin for another 3 weeks. No recurrence was observed.

## 4. Review of the Literature

A total of 89 articles were retrieved from literature search, of which 6 [2, 3, 7–10] reported 16 cases of acute clavicle osteomyelitis in children and adolescents (ages ranging between 0 and 16 years) that were included in the analysis. We excluded chronic cases of clavicle osteomyelitis and those for which the age and gender of patients were not reported. In total, 16 patients (56% female) were included in these studies, with a median age of 9 years ranging between 2 wks and 16 yrs. In all 16 cases local pain was the main presenting finding, whereas in 4 cases there was additional swelling and in 5 cases there was fever. In 9/16 (56%) cases the affected site was the right clavicle. Among these 16 cases,* S. aureus* was the most common organism (6 cases, 60% of 10 cases that had culture results reported) isolated from either blood or tissue. A microorganism had not grown in 3 cases with culture results reported, in 2 cases cultures had not been obtained, and in 4 cases had not been reported. Osteomyelitis appeared to be hematogenous in most of the cases with reported mode of dissemination. Duration of antimicrobial therapy varied, ranging from 4 weeks up to 3 months, with a median value of 7.5 years. Three patients needed surgical intervention: drainage 2 and curettage 1. Recurrence happened in 1/16 case and persistence of symptoms in 2/16 cases reported before 90s with unknown antimicrobial susceptibility of the pathogen.  Table 1 contains summary data of the published cases and our case.

## 5. Discussion

In this article we report a rare case of clavicle osteomyelitis in a child and review the previously published cases of acute clavicle osteomyelitis in children. Acute staphylococcal osteomyelitis of the clavicle is an infrequent but important entity. Before the introduction of antibiotics, acute hematogenous osteomyelitis was a serious disease with high morbidity and mortality. After the development of improved diagnostic tools and therapies, mortality from osteomyelitis in the developed world is negligible [11].

Our study shows that acute osteomyelitis of the clavicle tends to affect older children, as in our case, in which the patient was a 12-year-old boy. Recurrence happened in 1/16 cases and persistence of symptoms happened in 2/16 cases. The anatomy of the blood supply of the clavicle is a possible reason that treatment of acute staphylococcal osteomyelitis of the clavicle is difficult. With the nutrient artery entering laterally, pathologic fracture and disruption of blood supply may predispose to osteolysis of the medial segment. Osteomyelitis of the clavicle appears to behave like that of other flat membranous bones such as ilium, perhaps being more difficult to treat than osteomyelitis in long bones [2, 12].

The rarity of acute osteomyelitis of the clavicle and its clinical similarity to trauma result in frequent misdiagnosis. When a patient with acute focal pain and swelling at the clavicle is first seen, the clinical possibilities to consider include fracture, cellulitis, and soft tissue abscess. After consideration of trauma, the differential diagnosis of chronic osteomyelitis needs to weigh sternoclavicular arthritis, ischemic necrosis, developmental anomalies, and tumor or tumor- like conditions, such as eosinophilic granuloma, aneurysmal bone cyst, hemangioma, and osteoid osteoma. Because of its prevalence in children, Ewing sarcoma should always be considered when a child presents with local pain, swelling, and fever [7].

Our study showed that* S. aureus* is most commonly implicated (60%) and surgical drainage may be needed. While a variety of bacterial pathogens may be involved,* S. aureus* is the preeminent pathogen of acute hematogenous osteomyelitis infections in children [11]. Other causative agents include* Streptococcus pyogenes*,* Streptococcus agalactiae* (in infants),* Streptococcus pneumoniae*, coagulase-negative staphylococci (in device-associated infections),* Kingella kingae*, and enteric Gram-negative bacteria (especially* Salmonella* spp. in sickle cell patients) [1, 11].

MRI is the most appropriate method to evaluate bone marrow changes and it can detect osteomyelitis with sensitivity 82–100% and specificity 75–95% [13]. MRI is also useful for evaluation of complications such as abscesses, joint effusions, and soft tissue extensions that may require surgery [14].

Computed tomography (CT) shows the destruction of bones and detects complications such as abscesses, fistulas, or sequester formation. Comparing the two methods, MRI has a significant advantage to CT with regard to the imaging of the soft tissue involvement [15].

CT scan bears the burden of radiation; and availability of MRI/contrast administration issues remains. Ultrasonography could be a better alternative for the abscesses and soft tissue changes. It is a rapid, nonionizing, and very sensitive method for infectious fluid collections and joint effusions. Moreover, the images are not degraded by metallic or motion artifacts (as with CT and MRI) and finally ultrasonography offers the possibility of fine-needle aspiration to confirm the infectious nature of a fluid collection without unnecessary contamination of adjacent anatomical compartments [16]. Detection of osteomyelitis in our case was based on MRI findings (Figure 1(b)).

While patient symptoms and signs, laboratory findings, and diagnostic imaging are important, none of them is definitive with regard to a diagnosis of acute hematogenous osteomyelitis and, more importantly, none provides information on susceptibility of the challenging organism to antibiotics. For this reason, isolation of the etiologic organism remains the diagnostic gold standard and is currently the only way to establish a definitive microbiologic diagnosis [11, 17]. When the etiologic agent is identified, empiric antimicrobial therapy should be adjusted based on the specific susceptibility profile of the offending bacterial strain.

The successful treatment of all forms of osteomyelitis requires appropriate antibiotic therapy. Antibiotics that have proven efficacy against* S. aureus* bone and joint infections include antistaphylococcal penicillins, clindamycin, first- generation cephalosporins, and vancomycin [1, 17]. Harik and Smeltzer claim that, in communities with >10% CA-MRSA, vancomycin or clindamycin (if local clindamycin resistance rate is <25%) should be used as empiric treatment [18]. In our case, the choice of clindamycin led to a successful outcome.

Standard therapy for acute hematogenous osteomyelitis ranges from 4 to 6 weeks [19]. The review of cases shows a median value of 7.5-week duration. Evidence-based data about the route and duration of administration of antibiotics in acute hematogenous osteomyelitis are limited and criteria establishing when to switch from parenteral to oral therapy are undefined [19]. CRP typically becomes normal within a week of appropriate therapy and is frequently used as a marker for the switch from parenteral to oral therapy [1]. The oral antibiotic must demonstrate adequate bone penetration and have similar antibacterial spectrum and activity as the parenteral drug, and the little patient should be able to swallow oral medications [18].

In conclusion, acute clavicle osteomyelitis mainly affects older children and has generally good prognosis.* Staphylococcus aureus* is most commonly implicated and surgery may be needed.

## Figures and Tables

**Table 1:** Cases of acute clavicle osteomyelitis.

Number	First author/Publication year	Age	Gender	Symptoms & signs	Side	Isolate	Treatment	Outcome
1	Morrey/1977	2 wks	M	Painful swelling	Right	Hemolytic *Streptococcus*	IV penicillin 300,000 U × 210 days initially and then (due to abscess) IV penicillin 300,000 U × 6 for 2.5 weeks	Abscesses of right clavicle were incised and drained. IV penicillin 300,000 U × 6 for 2.5 wks, during which the patient became asymptomatic. 16 yrs later no recurrence
2	Morrey/1977	16 yrs	M	Pain and swelling	Right	*S. aureus*	Parenteral penicillin 500 mg × 4 for 17 d (due to drainage from the wound) Erythromycin (orally) 500 mg × 4 for 2 wks	One month after the abscess was drained, the patient was referred because of persistent drainage from the wound The patient was placed on erythromycin and the wound ceased draining during 2 weeks No recurrence after 9 yrs
3	Donovan/1982	12 yrs	F	Pain, swelling	Left	No growth	8-weak antibiotics	NR
4	Donovan/1982	10 yrs	F	Pain	Left	Not cultured	Antibiotics	Recurrence after an interval of over a year, then antibiotics
5	Donovan/1982	9 yrs	F	Pain	Left	No growth	3-month antibiotics	The swelling persisted after 6 months; radiographs revealed further expansion with a well-organised periosteal reaction; final diagnosis was acute to chronic osteomyelitis; following curettage, the localised swelling at the medial end of the clavicle has subsided-disease-free
6	Franklin/1987	15 yrs	M	Pain	Left	NR^*∗*^	Empiric antibiotic therapy	NR^*∗∗*^
7	Franklin/1987	3 yrs	F	Pain	Left	NR^*∗*^	Empiric antibiotic therapy	NR^*∗∗*^
8	Franklin/1987	9 yrs	F	Pain	Right	NR^*∗*^	Empiric antibiotic therapy	NR^*∗∗*^
9	Franklin/1987	9 yrs	F	Pain	Right	NR^*∗*^	Empiric antibiotic therapy	NR^*∗∗*^
10	Gerscovich/1994	7 yrs	F	2-month pain & fever	Right	*S. aureus*	NR	NR
11	Gerscovich/1994	11 yrs	F	2-weak pain & fever	Right	*S. aureus*	NR	NR
12	Gerscovich/1994	13 yrs	F	6-weak pain	Right	No growth	NR	NR
13	Gerscovich/1994	16 yrs	M	3-weak pain & fever	Left	Not cultured	NR	NR
14	Lowden/1997	7 yrs	M	4-day pain	Right	*S. aureus*	3-weak IV antibiotics + 3-weak oral antibiotics	6 weak after discharge pathologic fracture of right clavicle; 5 months after initial presentation, resorption of segment of clavicle
15	Lowden/1997	8 yrs	M	Pain & fever	Left	*S. aureus*	3-weak IV flucloxacillin + 4-weak oral flucloxacillin	No recurrence at 1 year
16	Our case	12 yrs	M	Pain, fever	Right	*S. aureus*	3-day IV vancomycin & cefotaxime 3-weak IV clindamycin & 3-weak oral clindamycin	No recurrence

NR: not reported

^*∗*^Only 2 of 16 cases of acute and chronic osteomyelitis reported had positive cultures

^*∗∗*^Surgery was required in 2 of 16 cases of osteomyelitis. Otherwise, outcome is favourable.
